# Financial Burden as a Potential Barrier to Vaccine Completion in Post-hematopoietic Stem Cell Transplant Patients in Japan

**DOI:** 10.7759/cureus.56842

**Published:** 2024-03-24

**Authors:** Taku Ogawa, Nobuyasu Hirai, Hiroyuki Fujikura, Natsuko Imakita, Kei Kasahara

**Affiliations:** 1 Department of Microbiology and Infection Control, Osaka Medical and Pharmaceutical University, Osaka, JPN; 2 Department of Infectious Diseases, Nara Medical University, Nara, JPN; 3 Department of Infectious Diseases, Hyogo Prefectural Amagasaki General Medical Center, Amagasaki, JPN

**Keywords:** catch-up immunization, public expenditure, vaccine, immunization, hematopoietic stem cell transplant

## Abstract

Introduction

The administration of routine vaccinations to patients following hematopoietic stem cell transplantation (HSCT) is highly recommended. However, studies examining reasons for not completing vaccination in post-HSCT patients are lacking.

Method

We reviewed the medical records of patients who sought vaccination following HSCT from January 2012 to December 2018 at the Center for Infectious Diseases, Nara Medical University.

Results

Information regarding patients' backgrounds, administered vaccines, and reasons for not administering recommended vaccines was collected for the study. Thirty-five patients (22 men and 13 women) with a median time from HSCT to the first visit of 25 months were enrolled. Vaccine coverage was highest for diphtheria, tetanus, and acellular pertussis (DTaP) at 89% (31 patients), followed by 23-valent pneumococcal, measles/rubella/mumps, and Japanese encephalitis at 71% (25 patients), 71% (25 patients), and 63% (22 persons), respectively. However, vaccine coverage for hepatitis B, 13-valent pneumococcal, and Hib was low at 26% (three patients), 11% (four patients), and 40% (14 patients), respectively. The reason for not completing the recommended vaccination series was not provided for most cases; however, the economic barrier was cited for all vaccines.

Discussion

This study identified several cases in Japan where individuals stopped completing post-HSCT vaccinations due to financial constraints. Larger-scale studies may be necessary in Japan in the future for further investigation.

## Introduction

As the efficacy of routine childhood vaccinations decreases, adult patients are thought to require revaccination following hematopoietic stem cell transplantation (HSCT) [1,2]. To implement the recommended vaccination regimen, patients must make numerous clinic visits over an extended period, imposing a significant burden on them. Moreover, some patients may be hesitant to receive vaccinations for childhood diseases as adults due to concerns about potential misunderstandings or the risk of reactivating graft-versus-host disease, leading to vaccine hesitancy. In contrast to pediatric patients, who benefit from public subsidy programs covering the costs of necessary vaccines (which can total over 160,000 JPY), there is a notable lack of adequate support systems to lessen the financial burden of vaccinations on adult recipients [3]. This makes post-HSCT vaccinations a particularly daunting prospect for patients. In this situation, the extent to which recommended vaccinations are administered and the reasons for any lack of vaccination remain largely unclear.

## Materials and methods

Nara Medical University Hospital, Nara, Japan, has an outpatient clinic specializing in vaccines, and the hematology department refers patients who have undergone HSCT for revaccination. The hospital performs 17-30 HSCT cases annually. Information on demographics, primary disease, HSCT type, and time since transplantation was collected following a retrospective chart review of post-HSCT cases referred to the vaccine clinic between January 2012 and December 2018. We also reviewed the vaccines available for post-HSCT patients at Nara Medical University Hospital (hepatitis B virus [HBV], *Haemophilus influenzae* type b [Hib], DTaP, tetanus toxoid [TT], pneumococcal conjugate vaccine 13 [PCV13], pneumococcal polysaccharide vaccine 23 [PPSV23], Japanese encephalitis [JE], measles virus [MeV], Rubella virus [RuV], and mumps virus [MuV]) and investigated the administered vaccines. To determine the reasons for nonvaccination, we further investigated patients who had not received HBV, DTaP, PCV13, PPSV23, JE, Hib, or live attenuated vaccines.

We provided a standard vaccination schedule to post-HSCT patients referred for vaccination in our vaccine outpatient clinic (Table 1), considering the types of vaccines available in Japan [4].

**Table 1:** Immunization recommendations for patients after HSCT at the Center for Infectious Diseases, Nara Medical University, Nara, Japan HSCT: Hematopoietic stem cell transplantation; GVHD: Graft-versus-host disease.

Vaccine type	Recommended dose	Schedule								Remarks
		0 month	1 month	2 months	4 months	6 months	7 months	12 months	13 months	
Hepatitis B	3	1st dose	2nd dose	-	-	3rd dose	-	-	-	-
*Haemophilus influenza* type b (Hib)	3	1st dose	2nd dose	3rd dose	-	-	-	-	-	-
Diphtheria, tetanus, acellular pertussis (DTaP)	3	1st dose	2nd dose	-	-	-	←	3rd dose	→	-
13-valent pneumococcal conjugate (PCV13)	3	1st dose	-	2nd dose	3rd dose	-	-	-	-	Available for adults aged 65 years and older since June 2014 in Japan
23-valent pneumococcal polysaccharide (PPSV23)	1	-	-	-	-	1st dose	-	-	-	-
Trivalent-inactivated poliovirus (tIIV)	3	1st dose	2nd dose	-	-	-	3rd dose	-	-	-
Japanese encephalitis	3	1st dose	2nd dose	-	-	-	-	3rd dose	-	-
Influenza (IIV4)	1/year	Annual administration of a single dose before the influenza season								-
Measles	2	If more than two years have passed since HSCT, GVHD is under control, and immunosuppressive drugs are not being administered, it is recommended to administer two doses at least one month apart.								-
Rubella	2									-
Mumps	2									-

Thus, this study used guidelines by Tomblyn et al. and Ullman et al. as its basis [1,2]. Furthermore, we investigated the presence of grade ≥III adverse events based on the Common Terminology Criteria for Adverse Events, version 5, [5] as far as the medical records were available. This study was approved by the Nara Medical University Ethics Committee (approval no.: 2526) and conducted in compliance with Japanese domestic law and the Declaration of Helsinki.

## Results

Background of the patients

In total, 35 patients (22 males and 13 females) who were referred for vaccination following HSCT from January 2012 to December 2018 were included in the study; the median age was 52 (22-70) years. Primary diseases included myelodysplastic syndrome in 10 patients, followed by acute myeloid leukemia (six patients), follicular lymphoma (five patients), acute lymphocytic leukemia (three patients), and aplastic anemia (three patients). Bone marrow and cord blood stem cell transplantations were performed in 12 and 23 patients, respectively. The median time from transplantation to referral was 25 months. Patient demographics are summarized in Table 2.

**Table 2:** Number of patients vaccinated with each vaccine and breakdown of reasons for nonvaccination HBV: Hepatitis B virus; Hib: Haemophilus influenza type b; DTaP: Diphtheria, tetanus, acellular pertussis; PCV13: 13-valent pneumococcal conjugate vaccine; PPSV23: 23-valent pneumococcal polysaccharide vaccine; tIIV: Trivalent-inactivated polio vaccine; JE: Japanese encephalitis vaccine; GVHD: Graft-versus-host disease. * Those who had not received two doses of each of the three vaccines were classified as "not vaccinated." Four individuals who received only two doses of the measles vaccine due to financial cost were included.

Vaccine type	Vaccinated	Not vaccinated	Reasons why the vaccine was not administered	Number of patients	Remarks			
HBV	9 (26%)	26 (74%)	Cost not affordable	4 (15.4%)	-			
			Died before vaccination	1 (3.8%)	-			
			HBV under treatment	1 (3.8%)	-			
			No record	20 (77%)	-			
Hib	14 (40%)	21 (60%)	Cost not affordable	4 (19.0%)	-			
			Vaccine not yet approved	1 (4.8%)	-			
			Died before vaccination	1 (4.8%)	-			
			No record	15 (71.4%)	-			
DTaP	31 (89%)	4 (11%)	Cost not affordable	2 (50%)	One of them was administered tetanus toxoid.			
			No record	2 (50%)	-			
PCV13	4 (11%)	31 (89%)	Age restriction at the first visit	29 (82.9%)	-			
			No record	6 (17.1%)	-			
PPSV23	25 (71%)	10 (29%)	Cost not affordable	8 (80%)	-			
			No record	2 (20%)	-			
tIPV	29 (83%)	6 (17%)	Died before vaccination	1 (16.7%)	-			
			Cost not affordable	1 (16.7%)	-			
			No record	4 (66.6%)	-			
JE	22 (63%)	13 (29%)	Cost not affordable	3 (23.1%)	-			
			Died before vaccination	1 (7.7%)	-			
			Adverse events of other vaccines	1 (7.7%)	-			
			No record	8 (61.5%)	-			
Measles, mumps, rubella	25 (71%)	10 (29%)*	Cost not affordable	4 (40%)	Steroids were being administered for GVHD.			
			No medical indication	3 (30%)	-			
			Died before vaccination	1 (10%)	-			
			Adverse events of other vaccines	1 (10%)	-			
			No record	1 (10%)	-			

Hepatitis B vaccine

Nine patients (26%) completed the three vaccination doses, and the other patients were unable to complete vaccination because of financial reasons (15%), ongoing treatment for active hepatitis B (4%), and death before vaccination (4%). The reason for the remaining 20 patients (77%) was not documented.

Tetanus, diphtheria, and pertussis vaccine

Thirty-one patients (89%) completed the DTaP vaccination. Of the remaining four patients, one (25%) received three doses of TT for economic reasons; one (25%) did not receive the vaccine due to economic reasons, and the remaining two (50%) were unsure about the reasons for not receiving the vaccine.

13-valent conjugate pneumococcal vaccine

Although our hospital recommends a series of PCV13 and PPSV23 vaccinations, only four patients (11%) were vaccinated with PCV13. In Japan, PCV13 was only available for children until 2014, which became available to adults after 2014 but only for those aged 65 years and above until May 2020. Therefore, three patients (9%) and 26 (74%) patients aged ≤65 years who were seen before and after 2014, respectively, were not vaccinated because of the age restriction. Of the 31 patients who were not vaccinated, six (17%) were uncertain about the reason for not being vaccinated. Notably, two of the four patients who were vaccinated requested the vaccination knowing that their age was off-limits for the indication.

23-valent polysaccharide pneumococcal vaccine

Twenty-five (71%) patients were vaccinated, and 10 (29%) were not vaccinated. Of the 10 patients who were not vaccinated, the reason for nonvaccination was the cost burden. All four patients who were vaccinated with PCV13 had received PCV13 and PPSV23 consecutively.

Japanese encephalitis vaccine

Twenty-two (63%) patients completed the three doses of the JE vaccine, whereas the remaining 13 (37%) did not wish to be vaccinated. Of the 13 who were not vaccinated, five patients had unknown reasons; the most common reason was the cost burden (3, 23%).

*Haemophilus influenzae* type b vaccine

Fourteen (40%) patients were vaccinated, whereas 21 (60%) were not vaccinated. Of those not vaccinated, four patients cited financial reasons for nonvaccination.

Live attenuated vaccines (measles, rubella, and mumps)

Twenty-five (71%) patients received two doses each of measles, rubella, and mumps vaccine; four (11%) received two doses of measles only; three (9%) were ineligible for vaccination because the time since transplantation was less than two years; and three (9%) did not wish to be vaccinated. Three patients were unable to be vaccinated with a live attenuated vaccine because of anxiety evoked by adverse events from other vaccines, self-interruption of hospital visits, and death before vaccination.

Adverse events

A relapse of graft-versus-host disease (GVHD) was observed in a 45-year-old woman with an underlying myelodysplastic syndrome who had undergone an unrelated cord blood transplant. The patient was referred at the 23-month post-transplant stage and had been receiving prednisolone 2.5 mg/day for GVHD since the initial visit. However, the hematologist had considered that the GVHD status was tolerable for vaccination. At the first visit, the patient simultaneously received the DTaP vaccine and trivalent-inactivated influenza vaccine (tIIV), which led to numbness in her fingers. The attending hematologist suspected a GVHD flare-up, and the vaccination was suspended.

## Discussion

The vaccination coverage by vaccine type following HSCT demonstrated the following differences: 89% of patients were vaccinated against the DTaP vaccine, and >70% were vaccinated against PPSV23 and live attenuated vaccines, such as MeV, RuV, and MuV. In contrast, several patients did not desire to be vaccinated against HBV and JE because of their low prevalence and the possibility of reducing the incidence by behavior [6,7]. However, once HBV is contracted, covalently closed circular DNA remains in the hepatic cells for life and can cause de novo HBV, whereas JE can leave serious sequelae [8]. Recently, JE and HBV vaccinations have become increasingly important with frequent overseas travel, and catch-up vaccinations should be administered properly.

We have excluded the quadrivalent meningococcal conjugate vaccine (MCV4) from the recommended vaccines at our hospital. MCV4 has been available in Japan since 2014, and the recommendation at Nara Medical University should have been updated. However, the study period was completed without updating our recommendations because of the low nasal meningococcal colonization rate and incidence of invasive meningococcal disease (IMD) in Japan [9-11] and the cost being expensive at >25,000 JPY/dose. IMD has a high mortality rate and is prone to sequelae [12], so MCV4 will be added to our recommendation soon.

Of the vaccines we recommend, all but PPSV23 are routine vaccines for children in Japan. However, the complete cost of vaccinations for adults must be paid out-of-pocket, even after HSCT. Local governments in Japan primarily implement immunization programs. Based on a survey by the Ministry of Health, Labour and Welfare (MHLW) in 2018, only 5.2% (90/1741) of all local governments have a cost-assistance program for immunization. Furthermore, 39 of these 90 local governments in Japan have age restrictions on eligibility for subsidies, resulting in only 2.9% (51/1741) permitting adults to receive subsidies [13]. Our study is limited by its small sample size and the frequent ambiguity regarding the reasons for incomplete vaccination as per recommendations. Despite these limitations, our findings suggest the existence of instances where financial constraints necessitate the relinquishment of vaccination. Further research with larger cohorts is required to accurately quantify the prevalence of such cases and to elucidate the extent of financial barriers impacting vaccination completion.

Finally, we would like to discuss some of the limitations of this study. First, this was a single-center study with a small number of subjects, and because it was a medical record-based, retrospective study, there were several unknown reasons for not vaccinating the subjects. We contacted several people who were still attending hematology outpatient clinics at the time of our study and attempted to ascertain why they did not receive some of the vaccinations. However, none of them had a clear recollection of the reasons regarding whether they had given up some of their vaccinations. Therefore, we concluded that although our study included many cases in which the reasons for nonvaccination were unknown, it would be difficult to collect additional information.

Furthermore, we did not have explicit criteria for the order in which vaccines were omitted when some vaccines were eliminated because of the cost burdens. Notably, the number of HSCT cases managed by the hematology department significantly exceeded the number of cases referred to our center. Our data does not provide reasons for why some patients were not referred to us by their hematologists. Previous reports have suggested that social factors including anti-vaccination sentiment or vaccine hesitancy, financial burden, and increased number of visits to the hospital may be factors that inhibit the completion of vaccination [14]. However, because of the lack of documentation, our study could not delve into these aspects, indicating the need to conduct future prospective studies in this area. Finally, before being referred to our center by the hematologist, the patients were informed that the vaccination was at their own expense, so the patients may refuse to be referred at that point.

## Conclusions

Regarding the current status of vaccination following HSCT at the Nara Medical University, we could not conclude that the recommended vaccines were sufficiently administered. Despite the small sample size and unclear reasons for vaccine incompletion in many cases, it was obvious that at least some patients gave up the vaccine after HSCT due to the economic burden. Hence, conducting more comprehensive and large-scale studies in the future is recommended.
